# The Morphological, Behavioral, and Transcriptomic Life Cycle of Anthrobots

**DOI:** 10.1002/advs.202409330

**Published:** 2025-06-06

**Authors:** Gizem Gumuskaya, Nikolai Davey, Pranjal Srivastava, Andrew Bender, Léo Pio‐Lopez, Douglas Hazel, Michael Levin

**Affiliations:** ^1^ Allen Discovery Center at Tufts University Department of Biology Tufts University Medford MA 02155 USA; ^2^ Wyss Institute for Biologically Inspired Engineering Harvard University Boston MA 02115 USA; ^3^ Bioinformatics Department University of Michigan Ann Arbor MI 48109 USA

**Keywords:** biobots, repair, synthetic morphology, transcriptomics

## Abstract

Fascinating aspects of morphogenetic and behavioral plasticity of living material are revealed by novel constructs that self‐construct from genetically wild‐type cells. Anthrobots arise from cultured adult human airway epithelial cells, developing, becoming self‐motile, and acquiring neural repair capabilities without exogenous genetic circuits or inorganic scaffolds. Progress in bioengineering and regenerative medicine depends on developing a predictive understanding of collective cell behavior in novel circumstances. Toward that end, here a number of life cycle properties of Anthrobots, including their morphogenesis, maturation, and demise, are quantitatively characterized. A self‐healing capacity and a remarkable reduction of epigenetic age upon morphogenesis are uncovered. Transcriptomic analysis reveals that assembling into Anthrobots drives a massive remodeling of gene expression relative to their cellular source, including several embryonic patterning genes, and a shift toward more evolutionarily ancient gene expression. These data reveal new aspects of engineered multicellular configurations, in which wild‐type adult human cells self‐assemble into an active living construct with its own distinct transcriptome, morphogenesis, and life history.

## Graphical abstract

DNA is thought to determine shape, behavior, and lifespan, with developmental plasticity reserved for stem cells, embryos, and species like amphibia. How much morphogenetic plasticity do adult somatic human cells exhibit? Anthrobots are a self‐organizing, motile synthetic living construct made of genetically normal human epithelial cells. They are evolutionarily novel, with self‐healing, age‐reversal, and a radically different gene expression profile.

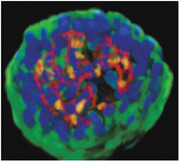

## 1 Introduction

Biobots have significant potential to impact discovery in biology, biomedical engineering, and biomedicine, emerging as a unique blend of biological and robotic concepts. Their relevance broadly falls across 3 categories. First, they contribute to basic evolutionary, cell, and developmental biology by revealing the developmental plasticity of cellular and tissue‐level morphogenesis in novel contexts, which are not apparent from stereotypical wild‐type development. Second, they can be used as a sandbox for bioengineering, active matter, and synthetic morphology efforts to understand the rules of self‐assembly of living materials for creation of novel synthetic living machines.^[^ ^1^ ^]^ Finally, they show promise as a technology for sensing and delivery of stimuli and cargo in vivo, in biomedical settings.^[^ ^2^ ^]^

Biobots can be categorized into three types: noncellular biobots, hybrid biobots, and fully cellular biobots.^[^ ^3^ ^]^ Noncellular biobots are entirely robotic but inspired by biological forms or behaviors. Examples include Wehner et al.’s fully autonomous soft “octobot,”^[^ ^4^ ^]^ Ze et al.’s origami millibots,^[^ ^5^ ^]^ Dillinger et al.’s micrometer‐scale ciliary biobots,^[^ ^6^ ^]^ Miskin et al.’s electromicroscopic robots,^[^ ^7^ ^]^ and several others^[^ ^7^, ^8^ ^]^ that can be controlled via magnetic, ultrasonic, or electrochemical actuation. A prime feature of noncellular biobots is their reductionist design approach, which seeks full control over structure and behavior. Hybrid biobots, a combination of biological and inanimate components,^[^ ^9^ ^]^ are exemplified by Nawroth et al.’s 2012 tissue‐engineered jellyfish^[^ ^10^ ^]^ as well as Park et al.’s 2016 biohybrid ray.^[^ ^11^ ^]^ These and other following examples of biobots^[^ ^7^, ^9^, ^10^, ^11^, ^12^ ^]^ integrate specialized tissues (e.g., muscle) with inert scaffolds, e.g., Polydimethylsiloxane (PDMS) gels, leveraging this synergy to drive form and function. By contrast, fully cellular biobots do not use inanimate material to shape and support the structure and resulting function, instead relying solely on the inherent capacity of biological cells to autonomously adopt new forms and functions, such as self‐motility. The first examples of these fully cellular biobots were Blackiston et al.’s different types of Xenobots,^[^ ^13^ ^]^ self‐motile ciliated spheroids derived from frog embryo cells. These bots were followed by Gumuskaya et al.’s first fully cellular human‐derived nonembryonic biobots in 2023, dubbed “Anthrobots.”^[^ ^3^ ^]^

Anthrobots are self‐constructing fully cellular biobots derived from human tracheal epithelia by reprogramming the development of an airway organoid. Airway organoids are 3D structures grown in vitro that mimic the architecture and function of the human airway.^[^ ^14^ ^]^ These organoids can derive from multiple sources, including stem cells, normal human bronchial epithelial cells (NHBEs), and human donors. Airway organoids are commonly characterized by their ability to replicate the cellular diversity of the airway epithelium, including ciliated cells, goblet cells, and basal cells. They exhibit key functional properties of the airway, such as mucus production, ciliary beating, and response to external stimuli, making them accurate models for studying respiratory physiology and pathophysiology—or, in our case, ciliated synthetic biological constructs. The main difference between Anthrobots and airway organoids is that while the primary goal of the organoids is to recapitulate the native tissue architecture and physiology, Anthrobots and other biobots demonstrate new living form and function that is not explored by evolution but is useful for engineering purposes. They are used not only to make useful synthetic living machines but also to explore motility‐enabled behavior that reveals plasticity and emergent features not apparent in organoid assays but may be of relevance to studies of evolution of behavior in basal forms of life and the emerging field of diverse (nonneural) intelligence.^[^ ^15^ ^]^

During maturation, various environmental cues can guide airway organoids toward certain morphologies, of which one of the most relevant is the topology of the cell's apicobasal polarity; this is important because active structures known as cilia form on the cells’ apical surface, and these can be oriented toward the inside of the sphere or the outside. By maintaining cell aggregates in growth media and Matrigel, airway organoids will develop a membrane with its apical (ciliated) surface facing inward. However, an apical‐out phenotype has recently been shown, resulting in cilia covering the exterior of the organoids. This phenotype has been accomplished using a variety of techniques, including air–liquid interfaces (ALI) with cells embedded in collagen,^[^ ^16^ ^]^ microwells that mold individual cell aggregates,^[^ ^17^ ^]^ U‐bottom wells without any extracellular matrix present,^[^ ^18^ ^]^ as well as in the case of Anthrobots, by way of sudden environmental phase shift from a semisolid to a liquid state.^[^ ^3^ ^]^ All these different approaches can trigger bronchial epithelium samples from humans to spontaneously form ciliated spheroids with self‐motility.

Anthrobots acquire shapes and behaviors quite different from any stage of human development or organogenesis; thus, they represent a fascinating opportunity to study the form and function of a new living configuration that had never been the subject of evolutionary selection in this form factor. Learning to predict and exploit emergent functionality of cellular materials is of critical importance to developing rational control in bioengineering and regenerative medicine contexts; Anthrobots are a new model system in which our nascent ability to predict novel life functions can be improved, specifically using an adult human cell source to study how much of the plasticity remains and can be functionally recovered in nonembryonic, complex life forms. Such synthetic examples of life are also interesting model systems from the perspective of evolutionary developmental biology and exobiology, as they are a unique context in which to understand “life as it could be,” as they differ from any known naturally evolved organism. Finally, because their morphogenesis and behavior are emergent in the context of wild‐type human cells, and not under control of exogenous synthetic circuits, molecular reprogramming factors, or nanomaterials, they can teach us much about the plasticity of the molecular hardware encoded by the genome.

Past work^[^ ^3^ ^]^ characterized Anthrobots’ shape, motility, and most strikingly, ability to induce repair in neural wounds in vitro. However, many aspects of their life history remained unknown, including the details of their formation process, the dynamics of their maturation and death, and their ability to respond to mechanical damage (regenerative capacity, as occurs in numerous organisms). While the bots come from adult (including elderly) donors, we wondered what their age was: could the process of morphogenesis reverse the clock? Epigenetic clock analysis^[^ ^19^ ^]^ revealed a significant reduction of age in the bots compared to their cellular source.

We also sought to characterize their transcriptome. While others study airway organoids as a model of human in vivo biology, we pursued a complementary perspective: as a new type of creature navigating an environment for which it was not directly selected, what genes would it express? We found massive alterations in the transcriptome of Anthrobots compared to their cells of origin, notably the upregulation of evolutionarily ancient genes. This reveals unexpected ways in which emergent morphogenesis regulates gene expression, including the appearance of embryonic patterning genes despite the adult origin of the cells and lack of genomic editing or transgenes. Taken together, these data shed light onto the natural history, at the molecular, cellular, tissue, physiological, and behavioral levels, of this novel synthetic architecture.

## 2 Results

### 2.1 Anthrobots Have Three Different Developmental Fates

**Figure 1:**
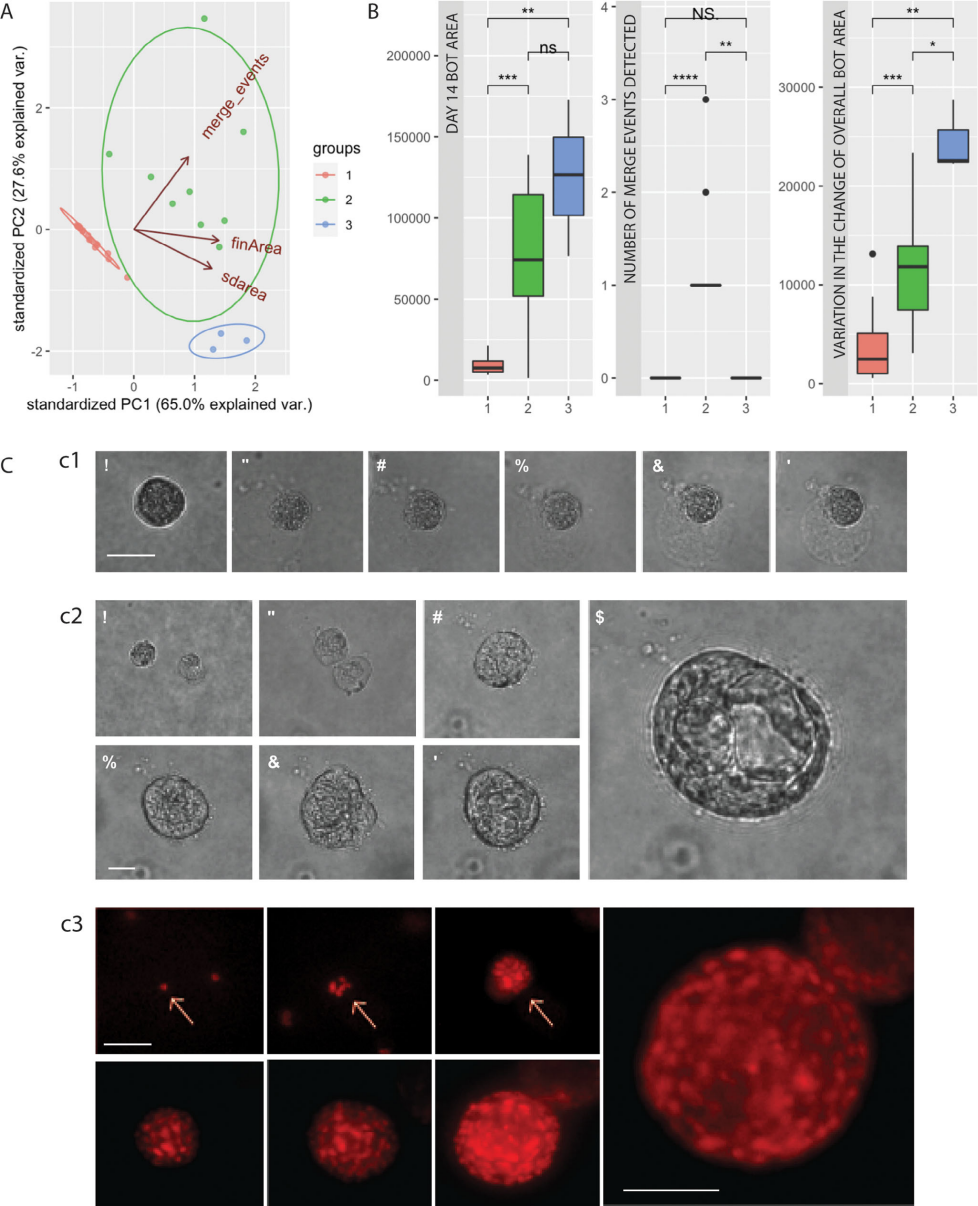
Anthrobots have different modes of self‐construction that may account for the emergent morphotypes. A) Principal components analysis (PCA) cloud consisting of 28 Anthrobots’ growth time course biomass data, evaluated with both morphological and temporal indices. The three orthogonal clusters identified by the unsupervised clustering algorithm are labeled as groups 1, 2, and 3, indicating three major fates respectively: dormant, merger expander, and monoclonal expander. The indices forming the PCA dimensions are as follows. The “merge_events” index is the master metric that distinguishes between Anthrobot formation via monoclonal expansion versus cluster merging: if a spheroid is undergoing monoclonal expansion, this number would be 1, while if the final spheroid is a result of multiple spheroids merging together, this number would denote the number of times a merge event has occurred. The “fin_area” measures the area of the final spheroid, while “sd_area” quantifies the average standard deviation of area across entire growth process. B) These three modes of growth are further described with the associated boxplots (*n* = 9 for group 2, *n* = 3 for group 3, and *n* = 16 for group 1). C) Representative examples from the PCA, as labeled on the cluster plot. c1) An example dormant bot. Notice volume stays the same. Scale bar 10 µm. c2) An example of hybrid Anthrobot formation through a merge event. Notice the heterogeneous structure in comparison to c1 and c3. Scale bar 10 µm. c3) An example monoclonally expanding, imaged bidaily. Scale bar 50 µm. *p*‐value range of 0–0.0001 corresponded to ****, 0.0001–0.001 corresponded to ***, 0.001–0.01 corresponded to **, 0.01–0.05 corresponded to *, and 0.05–1 corresponded to ns. All significance tests were evaluated at an alpha value of 0.05. Unless otherwise specified, the alternative hypothesis was always two‐sided for Wilcox tests.

**Figure 2:**
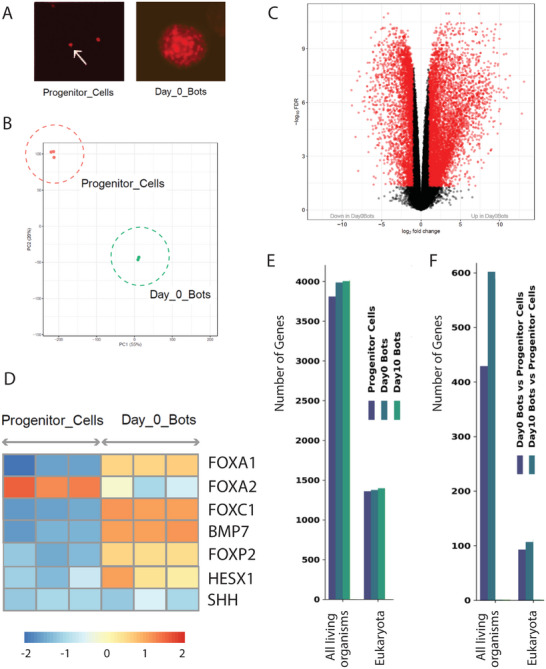
Early stage Anthrobots display onset markers of embryonic development. As NBHEs progress from progenitor cells to Anthrobots, we observe gene patterning characteristics of mammalian germ layer development and axis formation. A) Example morphologies of “progenitor cell,” at the beginning of Anthrobot growth time course, and “day 0 bot,” meaning it is on the last day of bot formation time course and day 0 of its spheroid (i.e., bot state) time course. B) PCA of clustering of Anthrobots across different life stages. Progenitor and day 0 bot stages are shown with dashed circles. C) The difference in gene expression between the progenitor versus day 0 stage, showing significant differences in the transcriptome, with notable genes explained further in panel (D) (*p* < 0.005; *n* = ≈1 million progenitor cells). D) Functional genes for germ layer formation (*FOXA1*, *FOXA2*, *FOXC1*, *BMP7*, and *FOXP2*) as well as axis formation (*BMP7*, *HESX*, and *SHH*) were observed to display expression profiles of early embryonic development. E) Histogram of the phylostratigraphic analysis of the Anthrobots and progenitor cells in the different conditions, showing the number of genes expressed with log counts per million (CPM) > 1 in the different conditions. F) Number of overexpressed DEGs with logFC > 2 in the different conditions. For (E) and (F), the *X*‐axis shows the evolutionary ages of ancient genes (here “All living organisms” and “*Eukaryota*,” the former corresponds to Eubacteria, bacteria, and their descendants). The *Y*‐axis shows the gene counts. See Supplement S1 in the Supporting Information for additional details. *N* = ≈1800 bots for day 0, 1000 bots for day 10, and 600 bots for day 25. *p*‐value range of 0–0.0001 corresponded to ****, 0.0001–0.001 corresponded to ***, 0.001–0.01 corresponded to **, 0.01–0.05 corresponded to *, and 0.05–1 corresponded to ns. All significance tests were evaluated at an alpha value of 0.05. Unless otherwise specified, the alternative hypothesis was always two‐sided for Wilcox tests.

**Figure 3:**
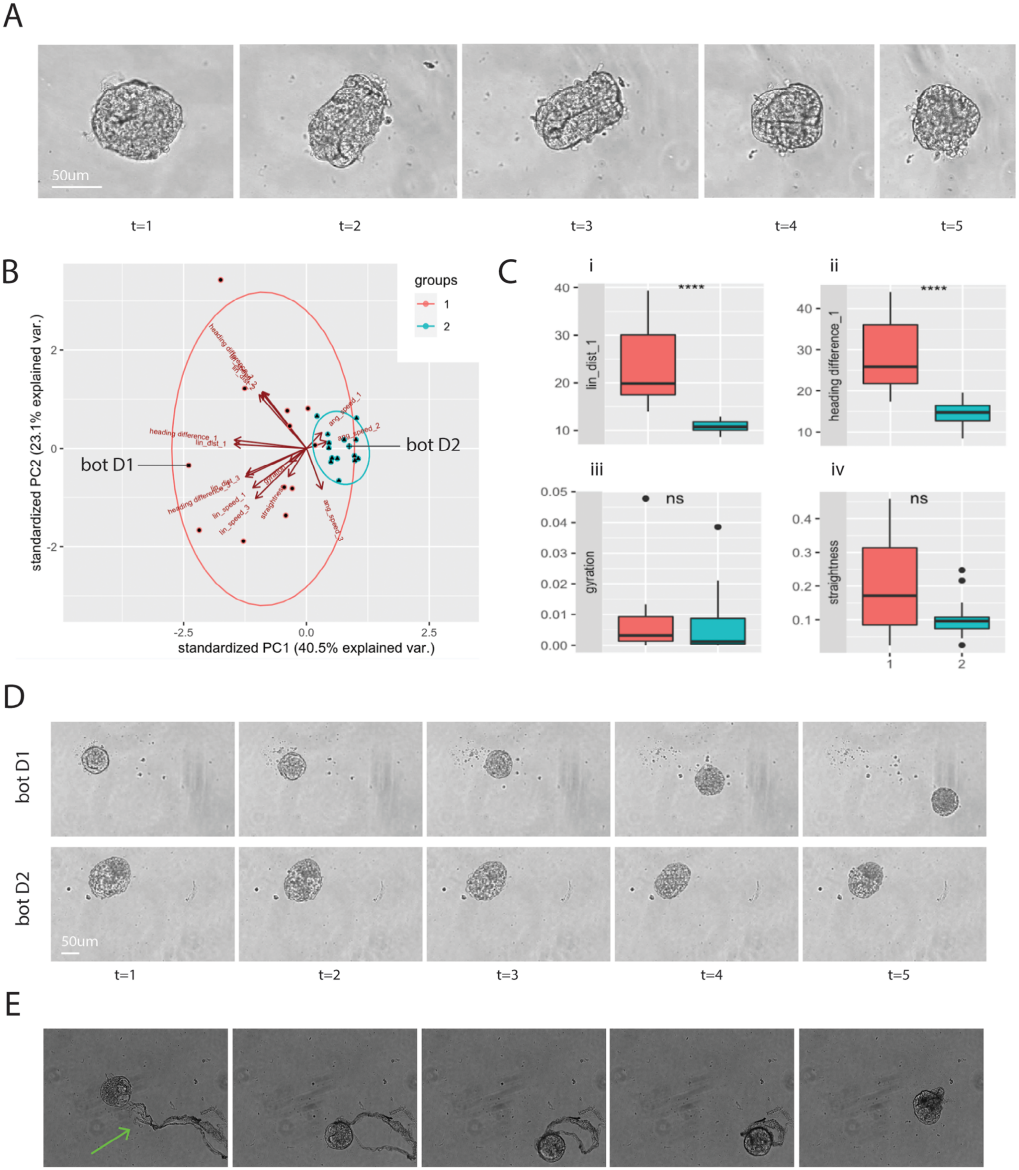
Anthrobots display different behaviors during the eversion process. A) Sample Anthrobot eversion time course. Day 0 Anthrobot going through polarity reversal via eversion across 48 h. B) PCA cloud consisting of *n* = 27 Anthrobots’ eversion time course biomass data, evaluated with both morphological and behavioral indices. The two orthogonal clusters identified by the unsupervised clustering algorithm are labeled as groups 1 and 2, indicating two major modalities of eversion. Plotting high‐dimensional cloud 2D. C) These two modes of eversion are further described with the associated boxplots (*n* = 12 for group 1, *n* = 15 for group 2). More specifically, while a majority of Anthrobots stay in place during eversion (group 2), which would be the expected behavior, a smaller subset of bots seems to be displacing during polarity reversal (group 1) as shown on graph i (*p* = 0.00027). This motility is likely caused by disorganized forces generated by radical anatomical activity, causing large variability in the heading of the displacing bots as shown in graph ii (*p* = 0.00018). The fact that there is no difference in terms of the metrics that measure organized behavior such as gyration (graph iii) or straightness (graph iv) between these two modes of eversion further supports this explanation. Straightness *p* = 0.051, gyration *p* = 0.8. D) Representative examples from each group across time. The individual bots are marked on the PCA cloud in panel (B) as botD1 and botD2. E) Collection behavior demonstrated by one of the motile eversion bots (group 2). The residual filament in cell culture media (shown with an arrow) is collected by a group 2 bot that ultimately encases the filament debris during eversion. Asterisks indicate statistical difference to *p* < 0.05. *p*‐value range of 0–0.0001 corresponded to ****, 0.0001–0.001 corresponded to ***, 0.001–0.01 corresponded to **, 0.01–0.05 corresponded to *, and 0.05–1 corresponded to ns. All significance tests were evaluated at an alpha value of 0.05. Unless otherwise specified, the alternative hypothesis was always two‐sided for Wilcox tests.

We first wanted to learn more about Anthrobots' self‐construction process and specifically investigated whether this process unfolds through a single path or whether there are different ways for Anthrobots to self‐construct as embedded in Matrigel, which may shine light onto their emergent morphological and behavioral differences.^[^ ^3^ ^]^ To this end, we collected microscopy images of 28 Anthrobots every 2 days to track their growth in Matrigel throughout a 14 days developmental process. We analyzed these time‐course data through three major metrics: number of cellular collisions (“merge_events”), final spheroid area (“fin_area”), and the average standard deviation of area across the entire growth process (“sd_area”), as shown in **Figure** **1**A. When this 3D data cloud was clustered with the WardD2 unsupervised clustering algorithm, we observed the emergence of three statistically orthogonal (Figure 1B) groups, each representing an Anthrobot growth type, which we named as dormant, merger expander, and monoclonal expander. The dormant typology consists of progenitor cells that start out as individual cells and never proliferate and develop into a multicellular spheroid (Figure 1C,c‐1). The merger expanders consist of the spheroids that result from the merging of one or multiple spheroids, each expanding from single cells through cellular proliferation (Figure 1C,c‐2). Finally, the monoclonal expanders are multicellular spheroids that can be tracked to a single ancestral cell (Figure 1C,c‐3). These classes are statistically distinct (as determined by the unsupervised WardD2 algorithm, *p* < 0.01). Thus, we conclude that Anthrobots have three different developmental fates.

### 2.2 Anthrobots Exhibit a Highly Altered, More Ancient, Transcriptome Including Upregulation of Embryonic Patterning Genes

Anthrobots undergo morphogenesis into a unique functional form despite their completely wild‐type human genome. What genes do Anthrobots express, and how do they compare to those of their parent tissue (airway epithelium) or to human embryos? To understand the plasticity of cell groups in the absence of genomic editing, we next sought to characterize their transcriptome. We first performed RNA‐seq on progenitor cells and day 0 Anthrobots (**Figure** **2**A), and computationally identified differentially expressed genes (DEGs) that were unique to Anthrobots. Biological replicates of the samples clustered tightly, but showed very clear separation between the source cells and Anthrobots (Figure 2B). Remarkably, plotting the transcripts with a strict cutoff (log2 FC > 4) for significance revealed massive remodeling of the transcriptome (Figure 2C). Out of 22 518 transcripts, 8992 showed significant up‐ or downregulation. Thus, becoming an Anthrobot has a major effect on the expressed genetic information.

Anthrobots self‐assemble, but they are made of adult, not embryonic, cells. Does this process involve any transcriptional programs normally driving embryonic development? We applied pathway analysis through the Gene Ontology (GO) database^[^ ^20^ ^]^ and found that the “embryonic patterning” gene category was highly represented when Anthrobot DEGs were compared to transcriptomic data of a human morula. We found that 13.4% of the overexpressed genes in Day0 bots compared to progenitor cells are genes activated at the morula stage. In addition, we found an enrichment in “multicellular organism development” (*p* < 0.005). We conclude that the formation of Anthrobots involves the activation of some embryonic transcriptional programs.

While Anthrobots appear to be spherically symmetric, and their source cells originate from the endodermal lineage, the embryonic‐like transcriptional signature led us to ask whether they expressed any embryonic markers of axial patterning^[^ ^21^ ^]^ and germ layer formation^[^ ^22^ ^]^ (Figure 2D). We observed the differential expression of both endoderm‐driver genes like Forkhead Box A1 (*FOXA1*) and Forkhead Box A2 (*FOXA2*)^[^ ^23^ ^]^ as well as mesoderm drivers such as Forkhead Box C1 (*FOXC1*)^[^ ^24^ ^]^ and Bone Morphogenetic Protein 7 (*BMP7*)^[^ ^25^ ^]^ in the earlier phase of the developmental process from progenitor cells to a 3D Anthrobot precursor spheroid. Key genes associated with ectoderm formation, such as Forkhead Box P2 (*FOXP2*),^[^ ^26^ ^]^ were not yet upregulated. Furthermore, while *BMP7*,^[^ ^25^ ^]^ which is also a marker of dorsal–ventral (DV) patterning, as well as the Homeobox Gene Expressed in ES Cells (*HESX*),^[^ ^27^ ^]^ driver of anterior–posterior (AP) patterning, were seen to be upregulated in this stage, Sonic Hedgehog (*SHH*),^[^ ^28^ ^]^ driver of left–right (LR) patterning, remained silenced. These findings indicate that Anthrobots, although not initiated from embryonic cells, demonstrate some molecular hallmarks of embryonic development as they display transcriptomic signatures that align with endoderm and mesoderm induction, as well as DV and AP patterning during this initial developmental stage, while the ectoderm genes, along with a LR patterning marker continuing to stay downregulated.

Next, we wondered whether the global transcriptomic changes associated with Anthrobot formation were associated with any phylogenetic shifts. To understand the age distribution of DEGs in Anthrobots under various conditions, we conducted a phylostratigraphic analysis^[^ ^29^ ^]^ (see also Supplement S1 in the Supporting Information), which revealed important differences in the expression of ancient genes across conditions. We found that progenitor cells express 3812 and 1360 genes, respectively, in the evolutionary ages “All living organisms” and *Eukaryota*. For Day 0 and Day 10 bots, it is, respectively, 3987 and 1375, and 4004 and 1398 genes that are expressed in these two evolutionary ages. Thus, we observed an increase in the genetic expression for unicellular genes ranging from 175 to 209 genes in the category “All living organisms” age and from 15 to 38 in *Eukaryota* genes (see Figure 2E). In addition, we observed that this effect is even more pronounced when we analyzed the DEGs between the anthrobots and their progenitor cells. Indeed, in the “Day0 Bots versus Progenitor Cells” and “Day10 Bots versus Progenitor Cells” conditions, we observed, respectively, 429 and 602 overexpressed genes (logFC > 2) for “All living organisms” evolutionary age and 93 and 107 overexpressed genes (log fold change (FC) > 2) in the *Eukaryota* evolutionary age (see Figure 2F).

Thus, phylostratigraphic analysis reveals that in addition to their transcriptome becoming more similar to that of embryos (moving backward in an ontogenetic sense), they also shift toward more ancient gene expression (moving backward in a phylogenetic sense).

### 2.3 Anthrobots Display Different Modes of Eversion during Their Self‐Assembly and Morphogenesis

Anthrobots undergo eversion as they mature, which brings the cilia into an outward‐facing orientation.^[^ ^3^ ^]^ To better characterize this process (Figure 1A), we next investigated whether this process unfolds through a single behavioral pattern or whether there are different paths for Anthrobots to undergo eversion upon their dissolution from Matrigel. We collected time‐lapse microscopy images from 27 Anthrobots during the first 48 h upon their dissolution from Matrigel with the goal of ascertaining its behavior over its entire trajectory. We analyzed these time course data (**Figure** **3**A) through a principal components analysis (PCA) composed of 14 axes featuring major metrics: the mean, median, and standard deviation of the angular speed, linear distance, heading and linear speed, as well as the movement straightness and gyration. When this 3D data cloud was clustered using the WardD2 unsupervised clustering algorithm, we observed two statistically orthogonal (Figure 3B) groups, each representing a distinct Anthrobot behavior during the eversion process, which we called displacers and statics (Figure 3C). The main distinguishing factor between these two different modes of eversion is the fact that while one group (group#1: displacers) consists of bots that show some degree of motility during the eversion process (Figure 3D‐1), the other group (group #2: static bots) show no motility during this process (Figure 3D‐2). Displacement during eversion is a surprising behavior given Anthrobot motility is generated by ciliary activity, which only surfaces at the end of the eversion process. This unexpected behavior, which may be due to some bots’ ability to displace mid‐eversion, thanks to partial localization of ciliated cells in the bot surface could grant Anthrobots with new capabilities, such as efficient engulfment of agents of interest in the environment, (e.g., particles, filament, or debris), as shown in Figure 3E.

### 2.4 Late‐Stage Anthrobots Express Markers of Maturing Embryonic Development despite Their Adult Tissue Origin

**Figure 4:**
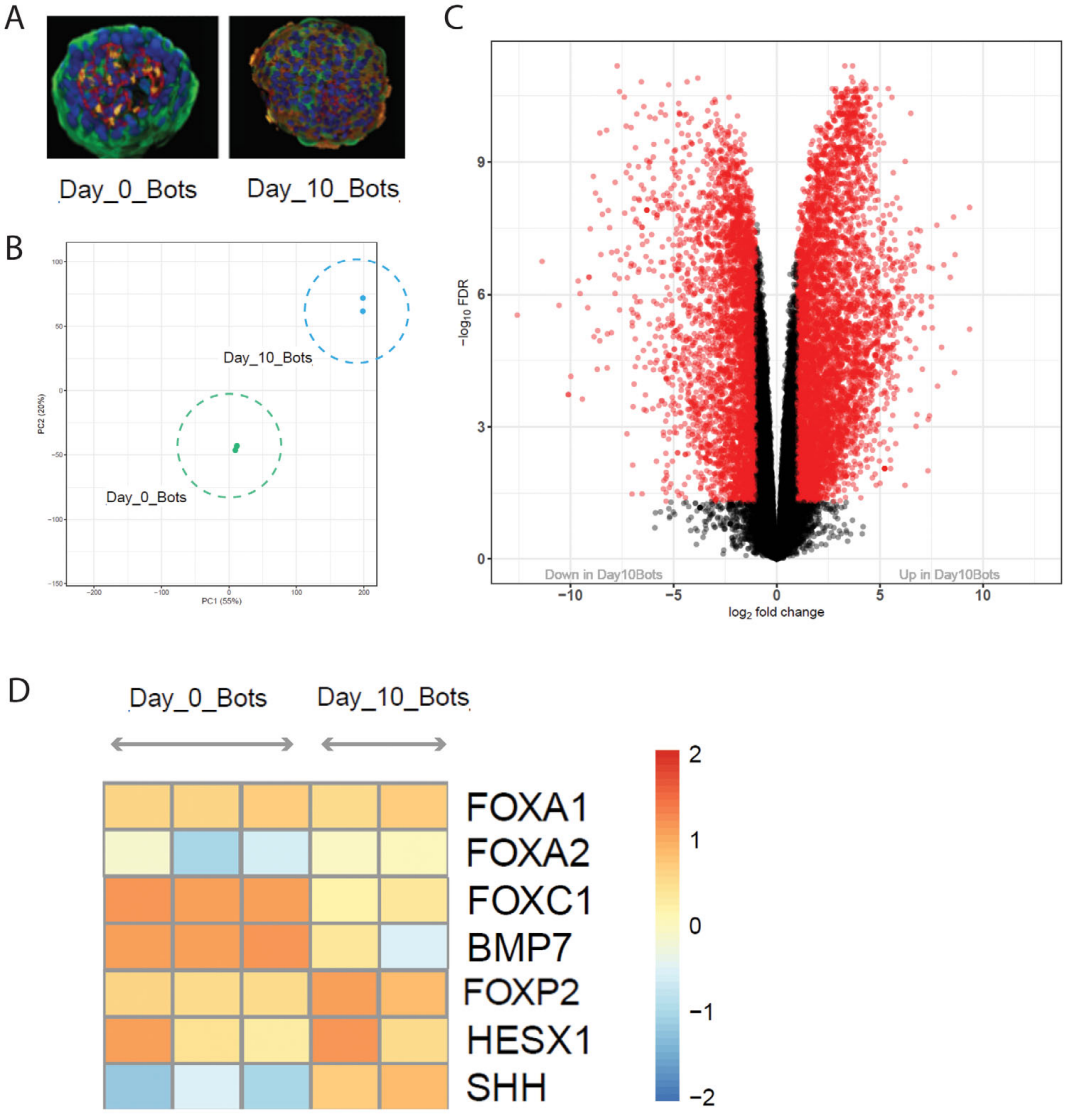
Late‐stage Anthrobots display markers of maturing embryonic development. As NBHEs progress from apical‐in state on day 0 to apical‐out state on day 10, we continue to observe gene patterning characteristics of mammalian germ layer development and axis formation. Colors green, blue, red, and yellow, respectively, represent basal cells, nuclei, tight junctions, and cilia. A) Example morphologies of “day 0 bot,” at the beginning of Anthrobot eversion time course, and “day 10 bot,” meaning it is at a mature motile state (*n* ≈1800 bots for day 0, *n* ≈1000 bots for day 10). B) PCA of clustering of Anthrobots’ transcriptome across different life stages. Day 0 and day 10 bots are shown with dashed circles. C) The difference in gene expression between the day 0 versus day 10 stage, showing significant differences in the transcriptome, with notable genes explained further in panel (D). D) *FOXA1* and *FOXA2*, key transcription factors for lung epithelial cell proliferation and differentiation in endoderm, show no difference of expression in day 0 versus day 10 bots. Similarly, genes like *FOXC1* and *BMP7* that are associated with mesoderm formation show decrease in expression. Conversely, an increase in the gene *FOXP2*, ectoderm marker, is seen as upregulated. Furthermore, for axis formation, while the dorsal–ventral patterning marker *BMP7* gets downregulated in this later developmental stage, the anterior–posterior marker *HESX* stays steady and the previously silent left–right patterning marker *SHH* becomes upregulated on day 10. *p*‐value range of 0–0.0001 corresponded to ****, 0.0001–0.001 corresponded to ***, 0.001–0.01 corresponded to **, 0.01–0.05 corresponded to *, and 0.05–1 corresponded to ns. All significance tests were evaluated at an alpha value of 0.05. Unless otherwise specified, the alternative hypothesis was always two‐sided for Wilcox tests.

Having characterized morphogenetic rearrangements in the maturing Anthrobots, we next sought to better understand the transcriptomic landscape of this synthetic self‐assembly process, as day 0 Anthrobots progress toward day 10 Anthrobots. RNA‐seq analysis (**Figure** **4**A) revealed significant differences (Figure 4B) that show significant changes (Figure 4C) in the expression of *FOXA1*, *FOXA2*, Fibroblast Growth Factor 8 (*FGF8*), *FOXP2*, Homeobox Gene Expressed in ES Cells 1 (*HESX1*), *SHH* genes – a further remodeling of gene expression past the initial changes compared to their tissue of origin (Figure 2). We conclude that Anthrobots undergo a second massive transcriptomic rearrangement, during their maturation.

Although the Anthrobots are made of adult human donor cells, they undergo significant morphogenetic processes, which raised the question of whether they expressed any genes associated with embryonic development despite their advanced age. The genes we were especially interested in were those involved in germ layer specification and establishment of primary body axes (Figure 4D). In this middle stage of Anthrobot development from day 0 to day 10, we observed no considerable change in the expression of endoderm‐associated genes such as *FOXA1* and *FOXA2*, as well as a downregulation of mesoderm‐driver genes such as *FOXC1*,^[^ ^24^ ^]^ *BMP7*.^[^ ^25^ ^]^ We did, however, find upregulation of the key genes associated with ectoderm such as *FOXP2* ^[^ ^26^ ^]^ in day 10 bots. Furthermore, *HESX11*,^[^ ^27^ ^]^ a gene involved in anterior–posterior differentiation, becomes further upregulated in day 10 Anthrobots as well. Likewise, a key regulator of left–right patterning and neurogenesis – *SHH* ^[^ ^28^ ^]^ – which had been silent throughout earlier stages of Anthrobot formation, also gets upregulated at this later stage. Taken together, these findings indicate that Anthrobots, although not initiated from embryonic cells, demonstrate molecular hallmarks of embryonic development as they mature, including transcriptomic signatures associated with the mesoderm to ectoderm transition, as well as anterior–posterior and left–right patterning.

### 2.5 Anthrobots Display Robust Behavioral Longevity and Reversal of Epigenetic Age

**Figure 5:**
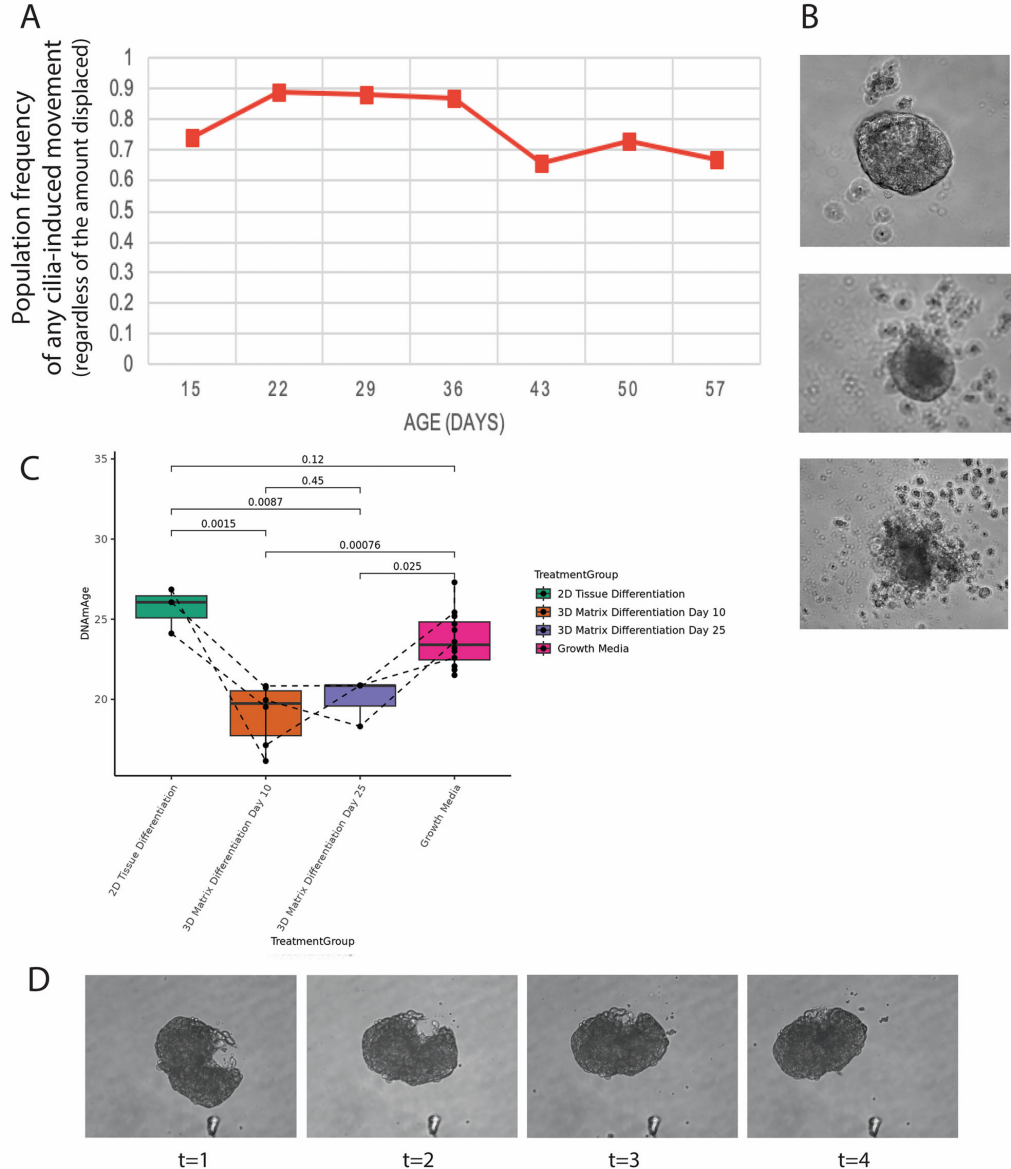
Anthrobots display robust behavioral longevity and reversal of epigenetic age. A) Weekly counts of baseline behavioral activity (defined as any cilia‐induced movement, regardless of the amount displaced) stay steady despite the shrinking population due to aging. B) Toward the end of an Anthrobot lifecycle, between weeks 4–8, while there is visible degradation of bot structural coherence (see Figure 6), the persistence of baseline behavior stays at a steady frequency. Population‐level baseline behavior outlives individual bots. C) Cells harvested from a 21 years old donor were differentiated into Anthrobots, which were then collected at day 10 and day 25 time points (*n* ≈ 1000 bots for day 10, and *n* ≈ 600 bots for day 25 time points). Methylation clock studies revealed passage 1 primary cells’ tissue age as 23.7 (mean value) years old (*n* = 12). Both against this baseline and against the recorded age of the donor, differentiation into Anthrobots introduced significant methylation clock reversal, with day 10 bots being read at the mean value of 19.1 years old (*n* = 6, *p* = 0.0015), while the day 25 bots being read at the mean age of 20.0 (*n* = 3, *p* = 0.0087). Contrarily, an air–liquid interface differentiation of the same cells into 2D airway tissue (ALI tissue) increased tissue age to a mean of 25.7 years old (*n* = 3, *p* = 0.0025 versus day 10 Anthrobot time point). D) Day 4 bot's healing process from a hypodermic needle injury (see Supplement S3 in the Supporting Information for additional details).

**Figure 6:**
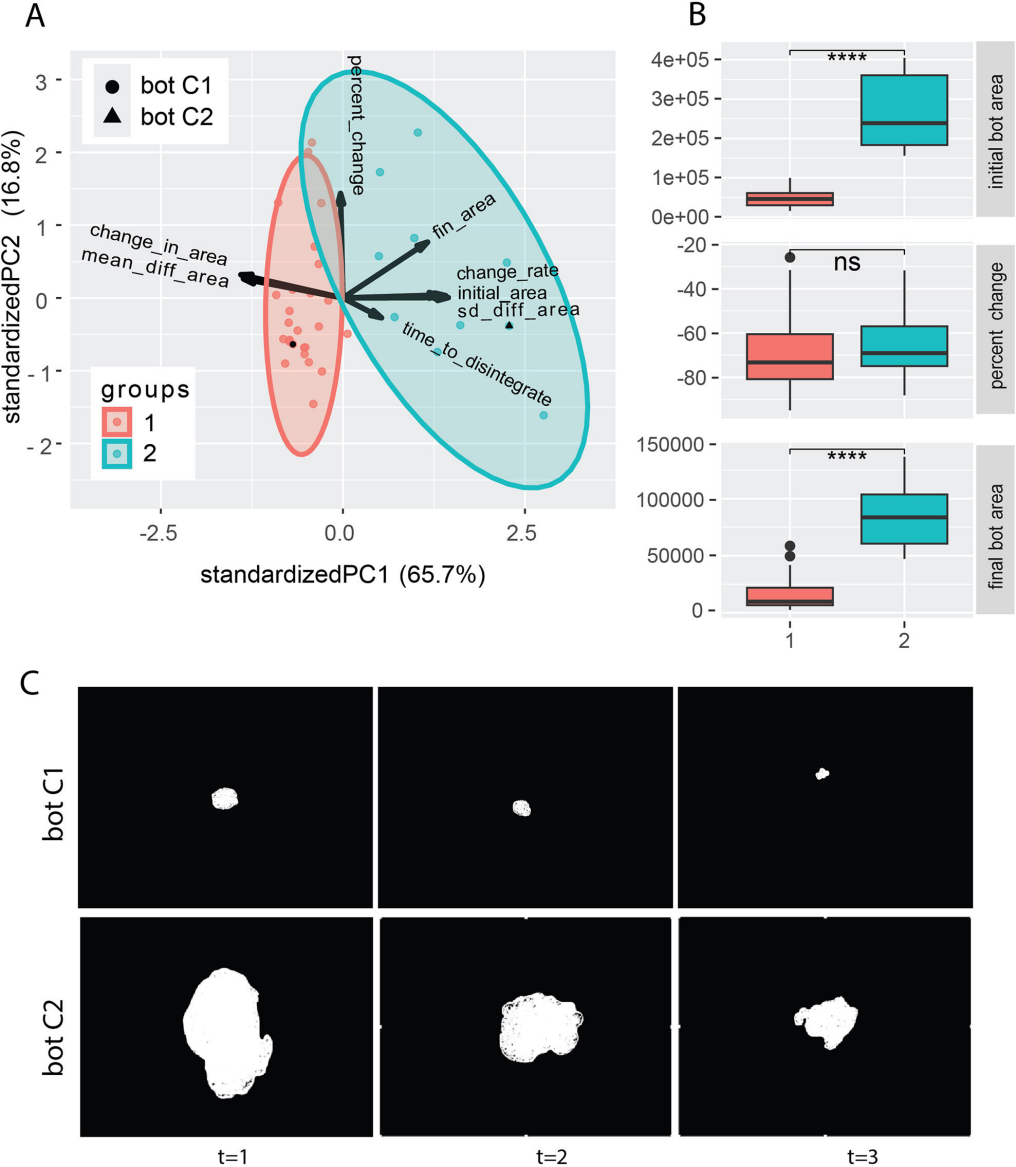
Regardless of initial size, Anthrobots degrade at a steady rate, resulting in large bots living longer. A) PCA analysis showing 36 bots’ cluster analysis based on morphological indices characterizing the change in size across a 30 days timeframe. B) Bots that start with a i) large initial area (group 2, *n* = 10) compared to bots that start with a smaller initial area (group 1, *n* = 26) show the same ii) relative percent change across their degradation time course, maintaining the size difference iii) at the end of the degradation process. Regardless of initial morphology, all Anthrobots are subject to a similar rate of aging, resulting in large bots living longer than smaller bots. Panels (i) and (iii) use arbitrary units in terms of pixel square. C) Bot C1 from group 1 (marked on the PCA) has a smaller initial area compared to bot C2 from group 2 with a larger initial area. While bot 1 completely degrades within the same time frame, bot 2 still stays intact, suggesting a positive correlation between bots’ initial morphology and their life span as opposed to a finite time span across all population. Scalebar 50 µm. Asterisks indicate statistical significance to *p* < 0.05. *p*‐value range of 0–0.0001 corresponded to ****, 0.0001–0.001 corresponded to ***, 0.001–0.01 corresponded to **, 0.01–0.05 corresponded to *, and 0.05–1 corresponded to ns. All significance tests were evaluated at an alpha value of 0.05. Unless otherwise specified, the alternative hypothesis was always two‐sided for Wilcox tests.

Having characterized the morphological and transcriptomic changes in developing and maturing Anthrobot populations, we next sought to characterize their aging profile. We collected weekly motility data from an arbitrarily chosen subset of Anthrobots to better understand behavioral profiles over time. The weekly counts of baseline behavioral activity, defined as any cilia‐induced movement, exhibited consistent levels (**Figure** **5**A) despite the declining population. Anthrobots approaching weeks 4–8 in their life cycle experience visible structural degradation (Figure 5B; refer to **Figure** **6** for a more comprehensive analysis). However, the frequency of motile behavior among intact bots at any given time remains steady, indicating bots do not cease their movement as they age, but only stop when they die – we found no evidence of an increasing elderly, fully immobile phase of life.

These living constructs underwent spontaneous morphogenesis and massive transcriptional remodeling with some embryonic signatures but were made of adult patients’ cells. What is their actual age? We decided to use human methylation aging clocks^[^ ^19^ ^]^ to better understand the impact of the Anthrobot life cycle on the age of the cells comprising them (Figure 5C). The experiment involved the use of cells from a 21 years old donor to create two sets of Anthrobots, aged 10 and 25 days, respectively. Methylation clock analysis was conducted to determine the tissue age of passage 1 primary cells, yielding a calculated tissue age of 25 years based on mean values. Remarkably, comparative analysis against the donor's recorded age and that of the cells revealed a substantial reversal in the methylation clock upon differentiation into Anthrobots. Specifically, the 10 days old Anthrobots displayed a mean age of 18.7 years, while the 25 days old Anthrobots exhibited a mean age of 20 years. These results suggest that the Anthrobots underwent a reversal in biological age – the process of development into bots reduced their epigenetic age by 25%.

### 2.6 Anthrobots Self‐Repair after Mechanical Damage

Finally, we asked whether Anthrobots had the capacity to repair after damage, and if so, given that this was not a normal human target morphology for any endogenous tissue or organ, what shape would they repair to? In order to test morphogenetic robustness, we damaged Anthrobots with a hypodermic needle and collected time‐lapse videos to characterize the response (Figure 5D and Video S1 (Supporting Information)). We observed that within the first 10–20 min of damage, the Anthrobots flex, and slowly return to the original shape they were in prior to the damage. The majority of structural recovery is owed to this immediate attempt to return to the original shape. Following this immediate damage recovery, we continued imaging the damaged bots through long‐term time‐lapse over the course of 48–72 h and observed no further “healing” or return to original shape beyond what they had recovered in the first 20 min. Furthermore, we observed that Anthrobots separated into two halves by the needle do not return to a single shape by the end of the 72 h; they remain distinct bodies. However, the initial flexing is present in the first 20 min postdamage.

Out of the 23 Anthrobots that were time‐lapsed immediately after being damaged, 16 showed recovery to their original spheroid shape, and 6 remained at an intermediate state (not fully indistinguishable from their original shape). One Anthrobot clearly did not show any recovery. Anthrobots showed no further recovery during a longer 72 h time‐lapse, and if they suffered a separation or a split during this period, they did not reform into a single whole but stayed in distinct pieces.

### 2.7 Regardless of Initial Size, Anthrobots Degrade at a Steady Rate, Resulting in Large Bots Living Longer

Anthrobots begin to degrade as they age, shrinking in size and losing displacement ability.^[^ ^3^ ^]^ To better characterize Anthrobot aging and degradation, we next investigated whether this process unfolds through a single behavioral pattern or whether there are different paths for Anthrobots to degrade. We collected time‐lapse microscopy images from 36 Anthrobots during the first 35 days upon their dissolution from Matrigel with the goal of characterizing their degradation rate. We analyzed these time course data (Figure 6A) through a PCA analysis composed of 8 major metrics: Initial area (area of bot in 1st frame), fin area (area of bot in final trackable frame), change in area (difference between fin area and initial area), percent change (change in area/initial area × 100), standard deviation of difference in areas (SD of change in area between successive frames), mean of difference in areas (mean of change in area between successive frames), time to disintegrate (number of frames until the bot becomes untrackable), and changerate (the maximum difference in the difference of areas, akin to the second derivative for the area). When this 3D data cloud was clustered using the WardD2 unsupervised clustering algorithm, we observed two statistically orthogonal (Figure 6B) groups, revealing a positive correlation between bots’ initial morphology and their life span. Regardless of initial morphology, all Anthrobots are subject to a similar rate of degradation, resulting in large bots living longer than smaller Anthrobots (Figure 6C). This reveals that Anthrobot initial morphology has a significant impact on Anthrobot longevity, as opposed to there being a definite life span across the entire population.

## 3 Discussion

Here, we focused on investigating the morphological, behavioral, and transcriptomic life stages of Anthrobots. We started by examining the self‐construction process of Anthrobots: as they mature, Anthrobots undergo eversion, bringing the cilia into an outward‐facing orientation. We found three distinct paths by which Anthrobots can self‐construct when embedded in Matrigel, illustrating a degree of variability/individuality in their morphogenesis. We also found that Anthrobots can recover their functional form after drastic mechanical injury (consistent with data in spheroids made from neonatal keratinocytes^[^ ^30^ ^]^). The fact that they self‐repair is likely an emergent feature of generic epithelial dynamics,^[^ ^31^ ^]^ because Anthrobots were never part of an evolutionary process in which ability to repair themselves was selected for, which impacts our understanding of the evolution of traits such as regeneration.^[^ ^32^ ^]^ This self‐repair process could make them a useful model for screening healing interventions or investigating the differences in self‐repair from cells of donors of different ages.^[^ ^33^ ^]^ Moreover, it has relevance to studies of mechanisms of epithelial closure,^[^ ^34^ ^]^ and also bodes well for the use of such biobots in vitro or in bioengineering settings, as they would not only be somewhat robust to damage from their environment, but could also positively affect nearby tissues via any secreted molecules that promote their own healing (as shown previously in their effects on neural scratch wounds^[^ ^3^ ^]^).

A key question in evolutionary developmental biology concerns the relationship between the molecular‐genetic and morphobehavioral information, and possible bidirectional control loops between them. Given that gene expression is under strong selection forces by a history in a specific environment, what would the transcriptome of a motile, self‐assembling novel living construct look like? We explored the genes expressed by Anthrobots and compared them to those of their parent tissue (airway epithelium) or human embryos to understand the plasticity of cell groups in the absence of genomic editing. We found that Anthrobots exhibit a much‐altered transcriptome and display embryonic markers, especially genes involved in axial patterning (consistent with other studies in the field, including nonhuman and salivary gland models^[^ ^35^ ^]^). Future studies using spatial transcriptomics will be needed to determine whether cryptic tissue‐level polarity or other kinds of biochemical patterning exist in the Anthrobots. More broadly, numerous kinds of synthetic constructs, such as more conventional scaffold‐based biobots,^[^ ^1^, ^12^, ^36^ ^]^ should be examined via mRNA‐ and protein‐level omics approaches to look for novel gene expression profiles induced by morphological configuration and behavior alone.

To understand the life cycle of this novel synthetic construct, we collected weekly motility data and behavioral profiles of aging Anthrobots; they displayed robust behavioral longevity, remaining motile until the very end of their lifespan, at which point they degrade by spontaneously dissociating. We investigated the aging and degradation of Anthrobots, finding that regardless of their initial size, Anthrobots degrade at a steady rate, resulting in larger bots living longer. Information on their end of life is important for potential in vivo uses of Anthrobots in patients for proregenerative applications.^[^ ^37^ ^]^ We also believe in vitro Anthrobots provide an attractive platform for screening antiaging candidate interventions, because they can be made patient‐specific, and because unlike traditional cell culture and organoid models, they offer motility, which can be used to quantify effects of putative prolongevity reagents not only on anatomical and molecular markers, but on active behavior. Ongoing studies will characterize that behavior with respect to ability to follow gradients (preferences) and ability to learn in simple assays.^[^ ^15^, ^38^ ^]^

The Anthrobots have a somewhat unique life history because the age of their cells does not match the age of their anatomical structure: they are made of adult (even elderly) cells, but their life as a multicellular creature begins anew. Anthrobots and similar systems stand to impact the field of longevity,^[^ ^39^ ^]^ as they reveal scenarios beyond the meiosis of embryogenesis that provide the reset needed to reverse aging.^[^ ^40^ ^]^ Thus, we sought to understand what impact their new morphogenetic configuration might have on cell‐level markers of aging, via an epigenetic clock approach;^[^ ^41^ ^]^ molecular clocks (and especially epigenetic clocks) have proven to be a valuable window on the aging process.^[^ ^41^, ^42^ ^]^ We found that fully assembled and motile multicellular Anthrobots are younger than their cells of origin. This is notable because it did not require any of the expected rejuvenation treatment and occurred in the absence of up‐regulation (forced or natural) of the Yamanaka reprogramming factors. What might have caused it? A similar event has been found in embryos,^[^ ^43^ ^]^ and it has been seen that senescence can be reduced by 3D culture.^[^ ^44^ ^]^

Our data with adult‐derived cells reveal that reduction of epigenetic age may not be an exclusive, programmed feature of embryonic development but could be generically induced by morphogenetic processes. One way to think about this is from the perspective of the cells as information‐processing agents which integrate signals from their internal and external environment. On the one hand, these cells had molecular components consistent with decades of adult life. On the other hand, all their current evidence – transcription of embryonic axial patterning genes, mechanical stresses of morphogenesis, etc., points to being part of an embryo. In the current protocol, this conflicting information induces a moderate roll‐back of the epigenetic clock (consistent with data^[^ ^45^ ^]^ on substrates that can return pluripotent stem cells to a naïve state). Our data are consistent with psychogenic contributions to aging,^[^ ^40b^ ^]^ in the sense that the physiological, morphological, and behavioral inputs during self‐assembly into a coherent 3D structure provide a kind of “age evidencing” that can propagate to other cellular age endpoints. To enhance this effect, with possible implications for the field of longevity, we suggest a research roadmap that exploits recent advances in extending neural decision‐making dynamics to understand cell responses.^[^ ^46^ ^]^ Frameworks such as active inference could be used to guide interventions to cell signaling^[^ ^47^ ^]^ to modify cells’ internal model of their own age.

There were a number of limitations in this study. Still unknown are metabolic profiles and consequences of this self‐assembly process. Likewise, the factors responsible for the individuality of the bots (the variety of their behaviors, life span, etc.) are unknown. With respect to behavior, at this point we do not know the rules governing their motion, but experiments in enriched environments with living and nonliving features will help to probe their behavioral repertoire and thus expand our understanding of basal cognition (especially in evolutionarily novel forms), and provide additional endpoints for screening of antiaging and nootropic compounds. Future work will also characterize Anthrobots’ biomechanical and bioelectrical properties, and the use of stimuli during their self‐construction process to guide form and function.

Synthetic living constructs made of the patient's own cells have the potential to impact biomedicine and in vitro organ bioengineering. Especially important is to augment studies of native biology in organoid models with ones that examine the emergent motile behaviors and nascent life histories, so that their properties and competencies can be predicted, exploited, and shaped toward useful applications. This technology is a positive contribution to active discussions of ethics in bioengineering and organoid research^[^ ^48^ ^]^ because they do not need to include brain tissue, are not derived from embryos, can be made from the patient's own cells with no heterologous animal material or nanomaterial components, and have a limited lifespan. This avoids some thorny issues that affect other related technologies.

By characterizing new ways in which adult somatic cells can reboot their multicellularity, and understanding the way these wild‐type cells navigate transcriptional, morphological, and behavioral spaces, novel aspects of the cell–body relationship are revealed. Novel transcriptomic profiles, driven by configurations and lifestyle, as well as behaviors not exhibited in the standard species‐specific morphological trajectory, have implications for basic evolutionary, cell, and developmental biology and the appearance of novelty and evolvability.^[^ ^49^ ^]^

## 4 Experimental Section

### Imaging for Material Growth (Growth of Bots in Matrigel with NLS)

The NHBE cells were seeded into a Matrigel suspension according to the previously established protocol.^[^ ^3^ ^]^ Once in the Matrigel suspension, select NHBEs (15–20 beacons/points of interest) were imaged using an EVOS M7000 (AMF7000) once every 3–4 days using a 20× objective in both Brightfield and Red Fluorescent Protein (RFP) until they had been 14 days in the Matrigel, at which point they were removed from the Matrigel suspension and used in downstream applications.

### RNA Extraction

RNA was extracted from Anthrobots and NHBE cells under different time points and experimental conditions. The extractions were performed using Invitrogen's TRIzol Reagent user guide as a reference. For the NHBEs, the only additional step was to trypsinize the cell layer with. 05% Trypsin for 3–4 min until the cells had been suspended in their media. Once the material (Anthrobots and NHBEs) was in suspension, it was collected into 15 mL conical tubes and centrifuged at 300 *g* for 3 min. The supernatant was then removed, and the material was resuspended in 1 mL of TRIzol and pipetted up and down to homogenize. Next, 0.2 mL of chloroform was added and then the sample was centrifuged for 15 min at 12 000 *g* and 4 °C. After centrifugation, the aqueous phase containing the RNA was transferred to a new tube. 0.5 mL of isopropanol was added to the aqueous phase and incubated at room temperature for 10 min. After incubation, the sample was centrifuged for 10 min at 12 000 *g* and 4 °C. The RNA formed a small, white pellet on the bottom of the tube. Next, the supernatant was discarded, being careful not to disturb the RNA pellet. The pellet was then resuspended in 75% ethanol, vortexed briefly, and centrifuged again for 5 min at 7500 *g* and 4 °C. The supernatant was then discarded, and the pellet was left to dry for between 5 and 10 min. After drying, 50 µL of RNAse‐free water was added to the pellet, and the sample was incubated on a heat block at 55 °C for 10 min. Finally, the RNA concentration in the sample was measured using a NanoDrop One from Thermo Fisher Scientific.

### rRNA Depletion RNA‐Sequencing

RNA quality was assessed via bioanalyzer at the Tufts Genomic Core, and Illumina Stranded Total RNA with Ribo‐Zero Plus was used for library preparation with high‐quality RNA. Upon library multiplexing, on Illumina HiSeq 2500, an rRNA depletion run using reversely stranded, single‐end sequencing was performed.

### Next‐Generation Sequencing (NGS) and Pathway Analysis

Reads were trimmed for adapter “GATCGGAAGAGCACACGTCTGAACTCCAGTCAC” and polyX tails, then filtered by sequencing Phred quality (≥*Q*15) using fastp.^[^ ^50^ ^]^ A count table was generated by aligning reads to the human transcriptome (Ensembl version 110) using kallisto^[^ ^51^ ^]^ and converting transcript counts to gene counts using tximport.^[^ ^52^ ^]^ To filter out low expressing genes, genes that had counts per million (CPM) more than 0.29 in at least 3 samples were kept. This CPM threshold was used so that genes that were expected to have at least 10 counts were kept, which was a rule‐of‐thumb value. There were 22 518 genes after filtering. Counts were then normalized by weighted trimmed mean of *M*‐values (TMM).^[^ ^53^ ^]^ If no normalization was needed, all the normalization factors would be 1. Here, the normalization factors were between 0.84 and 1.26. To use linear models in the following analysis, Voom transformation^[^ ^54^ ^]^ was performed to transform counts into logCPM, where logCPM = log2(106 × count/(library size × normalization factor)). Voom transformation estimated the mean–variance relationship and used it to compute appropriate observation‐level weights so that more read depth gave more weights. To get an overall view of the similarity and/or difference of the samples, PCA was performed.

### Phylostratigraphic Analysis of the DEGs

In order to achieve the phylostratigraphic analysis of the DEGs of the anthrobots in the different conditions, the evolutionary ages of all of the 19 660 protein‐coding human genes were first extracted from Litman et al.^[^ ^55^ ^]^ Litman et al. assigned these ages to 1 of the 19 major phylostrata defined by Domazet‐Loso and Tautz.^[^ ^56^ ^]^ These phylostrata were the following: all living organisms, (Eubacteria, bacteria, and their descendants), *Eukaryota*, *Opisthokonta*, *Holozoa*, *Metazoa*, *Eumetazoa*, *Bilateria*, *Deuterostomia*, *Chordata*, *Olfactores*, *Craniata*, *Euteleostomi*, *Tetrapoda*, *Amniota*, *Mammalia*, *Eutheria*, *Boreoeutheria*, *Euarchontoglires*, and *Primates*. The first two were selected to assess how much the anthrobots were behaving like unicellular organisms in terms of genetic expression and the number of genes expressed in the different conditions was counted.

### Comparison with *Morula* Genes

The list of morula genes was extracted from Yan et al.^[^ ^57^ ^]^ To do so, the genetic expression of each cell was averaged at the morula stage and only the genes with Reads Per Kilobase per Million mapped reads (RPKM) > 2 were kept. The overlap between the DEGs in the different conditions was computed with this list of morula genes. Then, the overlapped list of genes was enriched using g:Profiler^[^ ^58^ ^]^ (see also Supplement S4 in the Supporting Information).

### Imaging for Polarity Reversal Experiment

The imaging for the polarity reversal was done using Anthrobots recently dissolved from their Matrigel suspension in order to capture their inversion from apical‐in to apical‐out. The setup used involved a Bioscience Tools Stage Incubation system consisting of a CO_2_ Mixer (CO_2_‐MI), a top and bottom stage incubator (TC‐MWPHB), a temperature controller (TC‐1‐100i), and a humidifier (CO_2_‐500 mL). This was set up on an EVOS FL Auto microscope (AMAFD1000) used for all the imaging. After the imaging setup, the Anthrobots were removed from their Matrigel suspension following the protocol in the original Anthrobots paper (Motile Living Biobots Self‐Construct from Adult Human Somatic Progenitor Seed Cells). 200 µL of bronchial epithelial differentiation media (BEDM) was added to the inner wells of a 96‐well ultralow attachment round‐bottom plate from Corning (Cat no. 7007). The outer wells were filled with sterile DiH_2_O, which were found critical to prevent evaporation in the stage incubation setup. Once the plate was filled with media, the bots were moved to individual wells in a 96‐well ultralow attachment round‐bottom plate from Corning (Cat no. 7007). With one bot per well, the plate was placed into the stage incubation setup. The wells were imaged at 10× every hour for 72 h.

### PCA Clustering of Polarity Reversal Time Course

To find if any patterns in the behavior of bots during the polarity reversal stage could be computationally identified, Fiji was used to track the change in position of the bot for each frame using the AnalyzeParticles tool. After getting a coordinate analysis of each of the bots, a few variables were generated from the data. Initially, to determine the trajectory of the bot, the linear distance, speed, heading, and angular speed were estimated at each position to predict the coordinates of the following position. Of these variables, the mean, median, and standard deviation of the angular speed, linear distance, and linear speed were taken over the entire trajectory of the bot to try to ascertain its behavior over its entire trajectory. These 14 variables were part of the variables used to make the PCA. Two metrics were then derived to describe each trajectory: i) a “straightness” index computed as 1 minus the circular variance of the headings during the block (a value of 1 indicated a perfectly straight line) and ii) a “gyration” index computed as 1 minus the circular variance of the angular speed during the block divided by the circular variance of the same angular speeds and their additive inverse, which helped in taking into account the magnitude of the angular speeds themselves (a value of 1 indicated a trajectory following a perfect circle). These 14 variables were then used in a PCA, which had ≈63% variation in the first two components. Hierarchical clustering was then employed with the WardD2 method to derive two clusters.

### DNA Extraction

DNA was extracted from Anthrobots and NHBE cells using a QIAGEN DNeasy Blood and Tissue Kit with its protocol as a reference. All buffers and mixtures were provided by the kit, saved for phosphate‐buffered saline (PBS). For the NHBEs, the only additional step was to trypsinize the cell layer with 0.05% Trypsin for 3–4 min until the cells had been suspended in their media. Once the material (Anthrobots and NHBEs) was in suspension, it was collected into 15 mL conical tubes and centrifuged at 300 *g* for 3 min. The supernatant was then removed, and the material was resuspended in 200 µL of PBS. Then, 20 µL of Proteinase K was added to the material in suspension. Next, 200 µL of AL Buffer was added to the suspension and was mixed by vortexing. Next, 200 µL of 95–100% ethanol was added to the suspension, and the solution was again mixed by vortexing. The mixture was then pipetted into a spin column placed inside a 2 mL collection tube and centrifuged at 6000 *g* for 1 min. Then, after discarding the flow‐through, 500 µL of Buffer AW1 was added, and the column was centrifuged again at 6000 *g* for 1 min. Then, the flow‐through was discarded, and 500 µL of Buffer AW2 was added to the column. The column was then centrifuged for 3 min at 20 000 *g*; this centrifugation was repeated to dry the column. After the dry spin, the column was placed in a fresh 1.7 mL tube, and 200 µL of AE Buffer was added. It was let to sit for 1 min before centrifuging for a final time at 6000 *g* for 1 min. Afterward, the yield of the DNA collected in the 1.7 mL tube was measured using a NanoDrop One from Thermo Fisher and stored at 4 °C until needed for downstream applications.

### DNA Methylation Age Analysis

The DNA methylation array assays were performed using the following individual components. First was bisulfite conversion of extracted DNA using a Zymo EZ DNA methylation kit for downstream processing by either DNA methylation array technology. This was followed by MethylationEPIC BeadChip array kits, which included all the reagents required for DNA amplification, labeling, and array. The third step was to scan completed Beadchip arrays for the final DNA methylation readout. Finally, there was statistical analysis of measures of epigenetic aging for the respective species tested, an analysis of treatment versus control conditions, and an analysis of any available metadata for associations with changes to epigenetic aging. See Supplement S5 in the Supporting Information for additional details.

### Damage Experiment

Anthrobots were grown according to the previously established protocol^[^ ^1^ ^]^ and were transferred between days 2 and 11 into BEDM containing wells of a 96‐well ultralow attachment round‐bottom plate from Corning (Cat no 7007). The outer wells were filled with sterile DiH_2_O, which were found critical to prevent evaporation in the stage incubation setup. Next, bots were damaged with a hypodermic needle and upon withdrawing the needle, they were imaged immediately every 2 s for 10–20 min at 10× magnification using a Zeiss Axio Observer, equipped with an Axiocam 506 mono camera. The following longer term time‐lapses were acquired within a Bioscience Tools Stage Incubation system, consisting of a CO_2_ Mixer (CO_2_‐MI), a top and bottom stage incubator (TC‐MWPHB), a temperature controller (TC‐1‐100i), and a humidifier (CO_2_‐500 mL), using an EVOS FL Auto microscope (AMAFD1000). The wells were imaged at 10× every 30 min for between 48 and 72 h.

### Imaging for Degradation

The Anthrobots were imaged for their degradation in the following manner. First, the Anthrobots were dissolved from their Matrigel suspension according to the previously established protocol,^[^ ^3^ ^]^ then each well of a 96‐well ultralow attachment round‐bottom plate from Corning (Cat no. 7007) was filled with 200 µL of BEDM with Retinoic acid. After adding the media, Anthrobots were added individually to each well, resulting in 1 bot per well. The bots were imaged every 3–4 days at 20× in Brightfield using a Zeiss Axio Observer.Z1, equipped with an Axiocam 506 mono camera until they disintegrated to the point where no spheroid structure was visible. Media changes were performed every 4 days by aspirating 100 µL of media from each well and then adding 100 µL of fresh BEDM with Retinoic acid.

### Statistical Analysis

For all analyses, the Rstudio computational/statistical software was used.

### Statistical Analysis—Quantification of Anthrobot Growth Modalities

To computationally identify any patterns in the assembling of bots, Fiji was used to track the change in the size of the “bot area” for each frame using the AnalyzeParticles tool. After getting a CSV of each of the 28 bots, it was decided that experimental observations had three general categories needed to distinguish between 1) bots that senesced, i.e., died and stopped growing in size, 2) bots that grew completely from one cell to a bot and thus had no merges, and 3) ones that merged multiple cellular entities to form the final bot that grew to the final size. To distinguish these relationships, three variables were generated from the data, namely: i) merge_events: the number of times distinct spheroids merged to distinguish the bots that formed via mechanism (3) from those that formed via mechanisms (1) and (2); ii) fin_area: the final area of the bot at the end of the growing process which helped distinguish bots formed via mechanism (1) from those formed via mechanisms (2) and (3) since senesced bots ought to have significantly smaller area than other categories which grew to completion; iii) sdarea: the standard deviation of consecutive differences in area, i.e., the variation in the rate of change of area over time, which helped separate bots formed via mechanism (1) from those formed via mechanisms (2) and (3) since senesced bots ought to have smaller rate of change after it senesces (close to zero). This redundancy reinforced the results of the fin_area variable. These three variables were then used in a PCA, which had ≈90% variation in the first two components. Hierarchical clustering was then used with the WardD2 method to derive three clusters, which seemed to correlate in identity to the three types of bots to be distinguished.

### Statistical Analysis—Quantitative Identification of Degradation Clusters

In order to computationally identify any patterns in the bot degradation stage, Fiji was used to track the change in position, area, perimeter, and other physical parameters of the bot for each frame using the AnalyzeParticles tool. After getting a coordinate index of 36 bots, a few variables were generated from the data, focused mainly on deriving insights from the area of the main bot, so that change of area could tell about rates of degradation. These included initial area (area of bot in 1st frame), fin area (area of bot in final trackable frame), change in area (fin area − initial area), percent change (change in area/initial area × 100), SD of difference in areas (SD of change in area between successive frames), mean of difference in areas (mean of change in area between successive frames), time to disintegrate (frames till the bot is untrackable), and changerate (the maximum difference in difference of areas, akin to second derivative for area). These eight variables were then used in a PCA which had ≈82% of variation in the first two components. Hierarchical clustering was then used with the WardD2 method.

### Statistical Analysis—Pathway Analysis from RNA‐Seq

To discover the differential genes, limma was used, a R package that powers differential expression analyses.^[^ ^59^ ^]^ Linear modeling was performed and *t*‐tests were moderated to detect genes that were differentially expressed between groups. The GO database has hierarchical structures so that the GO terms can be grouped by their main (parent) categories.^[^ ^20^ ^]^ Gene Set Enrichment Analysis (GSEA) was performed in the GO database.^[^ ^60^ ^]^ Enriched GO terms were identified at a significance threshold of adjusted *p*‐value < 0.05.^[^ ^61^ ^]^ A network of the significant GO terms was constructed and the weights of edges were refined based on the Jaccard similarity.^[^ ^62^ ^]^ Pathway communities (i.e., clusters) were then identified by multilevel modularity optimization, i.e., Louvain algorithm.^[^ ^63^ ^]^ One GO term that had the smallest *p*‐value in GSEA was selected for each community. GSEA was performed in the Reactome database.^[^ ^64^ ^]^ Enriched pathways were identified at a significance threshold of adjusted *p*‐value < 0.05.^[^ ^61^ ^]^ A network of the significant Reactome pathways was constructed and the weights of edges were refined based on the Jaccard similarity.^[^ ^62^ ^]^ Pathway communities (i.e., clusters) were then identified by multilevel modularity optimization, i.e., Louvain algorithm.^[^ ^63^ ^]^ One Reactome pathway that had the smallest *p*‐value in GSEA was selected for each community. GSEA was performed in the Gene Transcription Regulation Database (GTRD) database.^[^ ^65^ ^]^ Enriched transcription factors were identified at a significance threshold of adjusted *p*‐value < 0.05.^[^ ^61^ ^]^ A network of the significant transcription factors was constructed and the weights of edges were refined based on the Jaccard similarity.^[^ ^62^ ^]^ Transcription factor communities (i.e., clusters) were then identified by multilevel modularity optimization, i.e., Louvain algorithm.^[^ ^63^ ^]^ One transcription factor that had the smallest *p*‐value in GSEA was selected for each community.

## Conflict of Interest

This work was supported by Astonishing Labs, which provides a sponsored research agreement to Tufts University and operates in the regenerative medicine space.

## Supporting information



Supporting Information

Supplemental Video 1

## Data Availability

The data that support the findings of this study are available from the corresponding author upon reasonable request.
