# Supplementary material for: The Morphological, Behavioral, and Transcriptomic Life Cycle of Anthrobots
Source: Adv Sci (Weinh). 2025 Jun 6;12(31):2409330. doi: 10.1002/advs.202409330 (PMC12376695; doi:10.1002/advs.202409330)
Supplement: Supplementary file 1 — Supporting Information [file ADVS-12-2409330-s001.docx]

Supplement 1: methodological details on phylostratigraphy and RNAseq

The human genes used in our phylostratigraphy analysis were obtained from the following list. By searching 13 publicly-available ortholog databases, Litman et al. [1] determined an estimate for the age of each of the protein-coding genes of the human genome and for a substantial fraction of the noncoding genes. Litman et al assigned these ages to 1 of the 19 major phylostrata defined by Domazet-Loso and Tautz [2]. They found the modal value of the earliest ortholog and defined this value as the age of that gene. For the 5 cases where no modal value could be found, they defined the age of the genes as the median value of the earliest ortholog across the searched databases.

RNA was extracted using ThermoFisher's TRIzol Reagent protocol, with approximately 1 million progenitor cells and six wells of Anthrobots, though the exact number of bots is difficult to accurately determine the precise count at both Day 0 and Day 10. The samples were sent to Tufts Genomic Core for RNA sequencing. Fig 4D follows a similar procedure, with the second condition involving Anthrobots collected 10 days after dissolution from Matrigel.

Supplement 2: video of Anthrobot healing wound

Supplement 3: methodological details on embryo degradation experiment

Anthrobots were imaged at 5X magnification, every 3-7 days, with shorter intervals as more bots began to degrade near the end of the experiment. The bots were kept in a 96-well u-bottom, ultra-low-attachment plate in order to prevent the bots from freely moving around the well, helping ensure they remain in the same field-of-view between imaging sessions. The FOV was saved in an onboard protocol on the microscope, a Zeiss Observer.Z1. The media was changed every 4 days, by removing 100uL of the old media and adding fresh 100uL of Bronchial Epithelial Differentiation Media with Retinoic acid (as in [3]) to the same amount. Counting of bots was done by eye, with those that had lost their spheroidal form and any ciliary motion considered fully degraded.

Supplement 4: methodological details on morula gene comparison

We applied over-representation analysis (ORA) for functional enrichment or gene set enrichment analysis (GSEA), on an input gene list [4]. It associates list of genes with established functional datasets (like Gene Ontology database [5] and identifies statistically significant enriched terms.

Supplement 5: methodological details on DNA methylation clock experiments

DNA methylation data was generated by the Epigenetic Clock Development Foundation (Torrance, CA) using Infinium MethylationEPICv2 BeadChip arrays (Illumina, San Diego, CA). This platform enables quantitative interrogation of over 930,000 CpG methylation loci per sample, covering all designable RefSeq genes, and including CpG Island shores, non-island CpGs, CpG islands outside coding regions, and miRNA promoter regions. The DNA methylation assays then involved bisulfite conversion of extracted DNA using a Zymo EZ DNA Methylation Kit, followed by DNA amplification, labeling, hybridization to MethylationEPICv2 BeadChip arrays, and scanning of the completed arrays for final DNA methylation readout.

Raw methylation signal intensities were obtained using the read.metharray.exp function from the minfi (v1.40.0) R package, followed by linear dye-bias correction and Noob background correction to address technical variation in background fluorescence signals [6]. Specifically, β-values were computed as the ratio of fluorescent signals from methylated and unmethylated sites, and these β-values were used in all analyses. Based on this data, several human epigenetic biomarkers of aging (epigenetic clocks) and estimated cell compositions based on blood methylation data were computed, including pan-tissue DNA methylation age (DNAmAge) [7], which was used for further analysis. DNAmAge was used as the primary measure of epigenetic age due to being a predictor of age across nearly all human cell types and tissues, including bulk tissue samples.

Cells for DNA methylation analysis were harvested from a 21-year-old donor before differentiation into Anthrobots, which were then collected at day-10 and day-25 timepoints, or alternatively into 2D airway tissue. Methylation clock studies assessed the following groups: passage 1 of primary cells tissue (n=12), bulk tissue after differentiation into Anthrobots and 10 days of culture (n=6); bulk tissue after differentiation into Anthrobots and 25 days of culture (n=3); and (d) tissue after differentiation of the progenitor cells into 2D airway tissue using an air-liquid-interface differentiation (ALI tissue).

An independent two-sample t-test was performed in R to assess differences between groups. Plots shown visualize the median values and interquartile ranges for each group, annotated with an asterisk for p-values < 0.05.

References Cited

[1] T. Litman, W. D. Stein, *Semin Oncol* **2019**, *46* (1), 3, <https://doi.org/10.1053/j.seminoncol.2018.11.002>.

[2] T. Domazet-Loso, D. Tautz, *BMC Biol* **2010**, *8*, 66, <https://doi.org/10.1186/1741-7007-8-66>.

[3] G. Gumuskaya, P. Srivastava, B. G. Cooper, H. Lesser, B. Semegran, S. Garnier, M. Levin, *Adv Sci (Weinh)* **2023**, e2303575, <https://doi.org/10.1002/advs.202303575>.

[4] a) L. Kolberg, U. Raudvere, I. Kuzmin, P. Adler, J. Vilo, H. Peterson, *Nucleic Acids Res* **2023**, *51* (W1), W207, <https://doi.org/10.1093/nar/gkad347>; b) A. Subramanian, P. Tamayo, V. K. Mootha, S. Mukherjee, B. L. Ebert, M. A. Gillette, A. Paulovich, S. L. Pomeroy, T. R. Golub, E. S. Lander, J. P. Mesirov, *Proc Natl Acad Sci U S A* **2005**, *102* (43), 15545, <https://doi.org/10.1073/pnas.0506580102>; c) W. Huang da, B. T. Sherman, R. A. Lempicki, *Nucleic Acids Res* **2009**, *37* (1), 1, <https://doi.org/10.1093/nar/gkn923>.

[5] M. Ashburner, C. A. Ball, J. A. Blake, D. Botstein, H. Butler, J. M. Cherry, A. P. Davis, K. Dolinski, S. S. Dwight, J. T. Eppig, M. A. Harris, D. P. Hill, L. Issel-Tarver, A. Kasarskis, S. Lewis, J. C. Matese, J. E. Richardson, M. Ringwald, G. M. Rubin, G. Sherlock, *Nat Genet* **2000**, *25* (1), 25, <https://doi.org/10.1038/75556>.

[6] M. J. Aryee, A. E. Jaffe, H. Corrada-Bravo, C. Ladd-Acosta, A. P. Feinberg, K. D. Hansen, R. A. Irizarry, *Bioinformatics* **2014**, *30* (10), 1363, <https://doi.org/10.1093/bioinformatics/btu049>.

[7] S. Horvath, *Genome Biology* **2013**, *14* (10), 3156, <https://doi.org/10.1186/gb-2013-14-10-r115>.
